# Midkine: A Novel Prognostic Biomarker for Cancer

**DOI:** 10.3390/cancers2020624

**Published:** 2010-04-20

**Authors:** Hirofumi Jono, Yukio Ando

**Affiliations:** Department of Diagnostic Medicine, Graduate School of Medical Sciences, Kumamoto University, 1-1-1 Honjo, Kumamoto, 860-8556, Japan; E-Mail: andoy709@kumamoto-u.ac.jp

**Keywords:** Midkine, tumor marker, cancer screening, prognostic prediction

## Abstract

Since diagnosis at an early stage still remains a key issue for modern oncology and is crucial for successful cancer therapy, development of sensitive, specific, and non-invasive tumor markers, especially, in serum, is urgently needed. Midkine (MK), a plasma secreted protein, was initially identified in embryonal carcinoma cells at early stages of retinoic acid-induced differentiation. Multiple studies have reported that MK plays important roles in tumor progression, and is highly expressed in various malignant tumors. Because increased serum MK concentrations also have been reported in patients with various tumors, serum MK may have the potential to become a very useful tumor marker. Here, we review and discuss the possibility and usefulness of MK as a novel tumor marker.

## 1. Introduction

Midkine (MK), a heparin-binding growth factor, was originally reported to be the product of a retinoic acid-responsive gene during embryogenesis [1,2]. In 1988, Kadomatsu *et al*. first isolated an MK cDNA clone by differential hybridization and reported that MK was intensely expressed in early differentiation stages of embryonal carcinoma cells [1]. Because the RNA was originally detected only in midgestation mouse embryos and the kidney in adults, MK was initially called “midgestation embryo and kidney (MK) gene” [2]. During the two decades since the discovery of MK, substantial advances have been made in understanding the biological activity and molecular basis of MK. It is well-documented that MK is a multifunctional peptide which, together with pleiotrophin (PTN), forms a structurally distinct family of heparin-binding growth factors [3]. Interestingly, despite its high expression during embryogenesis, MK is not detectable in healthy adults and only re-appears in the body as a part of the pathogenesis of diseases [3]. Moreover, the most intriguing feature of MK is its massive expression in advanced tumors with surprisingly-high frequency. In this review, we describethe molecular genetic and biological significance of MK, especially focusing on malignant tumor, and introduce the possibility of MK as a novel tumor marker.

## 2. Midkine

### 2.1. Molecular Genetic Characterization

**Figure 1:**
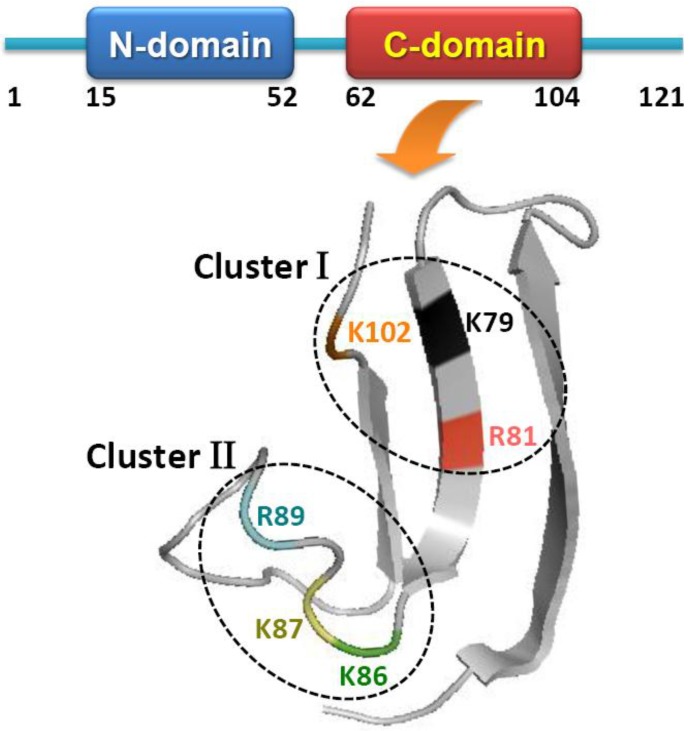
The domain structure of MK and the three-dimensional structure of its C-terminal domain.

The human midkine gene is located on chromosome 11q11 and encodes a 13-kDa protein rich in a basic amino acid and cysteine [4,5,6]. MK is widely conserved from *Drosophila* to human [7]. Human and mouse MK sequences are extremely highly conserved: 87% of amino acids are identical and all amino acid changes are conservative except for an insertion [8]. MK is composed of two domains: an N-terminally located domain (MK 15–52), and a C-terminally-located domain (MK 62–104) flanked by intra-domain disulfide bridges [9] (Figure 1). The three dimensional structure of MK has been clarified based on the structures of the N-terminal half and C-terminal half molecules determined by NMR [10]. In the C-terminal half of human MK, two heparin-binding clusters, namely cluster I (K79, R81 and K102) and cluster II (K86, K87 and R89), have been identified [10,11]. Cluster I is especially essential for the recognition of heparin sulfate as well as chondroitin sulfate proteoglycans, and responsible for multiple biological functions, such as neurite outgrowth, fibrinolysis, and nerve cell migration [11,12,13].

It is also well-documented that the expression of MK is developmentally regulated [1,2]. MK is highly expressed in the midgestation period in response to retinoic acid, whereas its expression becomes low or undetectable in normal adult tissues [3]. In the promoter region of the *MK* gene, there is a functional retinoic acid-responsive element, which is responsible for the MK expression induced by retinoic acid [14,15]. The 5’ regulatory region of the *MK* promoter also contains two binding sites for Wilms' tumor suppressor gene (WT1) [16]. The WT1-responsive element near the transcription start site of the *MK* promoter is required for reduction of MK expression by WT1. In addition, the transcriptional activity of the *MK* promoter is regulated by cell growth in part through p53-dependent pathways [17]. Recent studies also revealed that MK expression is regulated by hypoxia, cortisol, growth factors, and cytokines *via* a nuclear factor-κB (NF-κB) dependent pathway [18,19,20]. The precise regulatory mechanism underlying the tight regulation of MK expression remains to be elucidated.

### 2.2. Mechanism of Action

MK, a secreted plasma protein, initiates signaling through the ligand-dependent activation of receptors to participate in regulating diverse biological processes [7]. Several MK-binding cell surface proteins, including syndecans, integrins, protein tyrosine phosphatase ζ (PTPζ), anaplastic lymphomakinase (ALK), and low-density lipoprotein (LDL)-receptor-related protein (LRP) [7,21], have beenidentified. MK strongly binds to syndecan proteins, namely syndecan-1, -3, and -4 [22,23,24]. The binding of MK to syndecans is mediated by the heparan sulfate chains. PTPζ is a receptor-type protein tyrosine phosphatase of which the extracellular domain carries chondroitin sulfate chains, and the intracellular domain exhibits protein tyrosine phosphatase activity [7]. The chondroitin sulfate chains on its ectodomain of PTPζ are essential for MK-binding [13]. The interaction between MK and PTPζ activates phosphoinositide 3-kinase (PI3-kinase) and extracellular signal-regulated kinase (ERK) for osteoblast cell migration and neural survival [25,26]. ALK, a transmembrane tyrosine kinase, was identified to be a receptor for MK and PTN [27,28]. By binding of MK to ALK, PI3-kinase and ERK are activated for intracellular signaling and regulate cell growth [28]. In addition, LRP has been identified as a membrane protein, which was purified from the MK-affinity column [29]. LRP is a member of the LDL receptor family that accomplishes endocytosis of a diverse array of ligands [30]. LRP1 mediates MK endocytosis, and endocytosed MK enters the nucleus where it exerts anti-apoptotic activity [31]. Chen *et al*. reported that MK exhibits strong affinity for the N-terminal half of the second domain among the four ligand-binding domains of LRP1, and plays an important role in anchorage-independent cell growth [32]. Taken together, these receptors described above may regulate the biological activities of MK either independently or cooperatively. Further investigations are needed to specify the functional interaction and complex formation between these receptors.

### 2.3. Biological Significance

During the midgestation period of embryogenesis, MK plays important roles in the development of tooth, lung, kidney, bone and nerve tissues [33,34,35,36,37,38]. Morphogenesis and cell differentiation were inhibited in tooth germs cultured in the presence of neutralizing antibodies for MK [33]. In development of embryonic mouse lung, MK showed a weak effect on branching morphogenesis, but exhibited an effect in restoring development of mesenchymal tissue [34]. MK was also involved in kidney development *via* the molecular cascade of the epithelial conversion of the metanephric blastema [36]. In addition to the specific functions of MK in development, extensive evidence has also accumulated that MK has a huge variety of biological function, such as transformation, neural survival, tissue remodeling, cell growth, differentiation, cell migration, and carcinogenesis [3,7,19,39,40]. Kaneda *et al*. reported that MK exhibits nerve cell adhesion and guidance activity for neurite outgrowth *via* the heparin-like domain on nerve cell surface heparan sulfate proteoglycan [41]. MK, which has neuroprotective activity and neurite extension, expresses strongly cerebral infarct and Alzheimer’s disease [25,42,43,44,45]. After ischemic injury, MK is up-regulated by astrocytes in the surviving region of the cerebral cortex and the hippocampal CA1 subregion, suggesting that MK may contribute to the survival of injured neurons [43,46,47]. In the inflammatory response, MK promotes the cell migration of inflammatory cells, specifically macrophages and neutrophils [48,49]. In addition, MK expression increases at intraperitoneal adhesions after surgery and cardiac remodeling after myocardial infarction [50,51,52]. Most importantly, MK is significantly up-regulated in various malignant tumors and plays crucial roles in carcinogenesis [53,54,55,56]. Thus, due to its multifunctional properties, MK is deeply involved in the pathogenesis of various diseases, especially malignant tumors.

In accordance with its high expression in various malignant tumors, MK exerts cancer-related activities in the process of carcinogenesis, including transformation, fibrinolysis, cell migration, cell survival, anti-apoptosis, and angiogenesis [7,25,29,39,57,58,59,60,61]. Kadomatsu *et al*. reported that NIH3T3 cells were transformed by overexpression of MK, and formed tumors in nude mice [39]. MK serves as a mediator of retinoid and cooperates with basic fibroblast growth factor (bFGF) to enhance fibrinolytic activity of endothelial cells [57]. MK enhances the plasminogen activator (PA)/plasmin levels in bovine endothelial cells in a dose- and time-dependent manner, and the fibrinolytic activity can be achieved through this up-regulation of urokinase-type PA expression [57]. In addition, cell migration-promoting activity of MK has been demonstrated in neutrophils, osteoblastic osteosarcoma cells, neural cells, macrophages, and smooth muscle cells [13,38,40,49,58,62]. In primary neuronal cultures, MK inhibited caspase-dependent apoptosis *via* the activation of ERK and PI3-kinase [25]. Qi *et al*. also reported that MK rescued Wilms' tumor cells from cisplatin-induced apoptosis by regulating Bcl-2 expression [63]. Furthermore, angiogenic role of midkine has been shown by the observation that MK transfection into the breast carcinoma line, MCF-7, accelerated tumor growth, and increased tumor vascularity after implantation of the cells in nude mice [64]. Taken together, these distinct observations not only bring new insights into the novel biological roles of MK, but also open up novel therapeutic targets for the various malignant tumors.

## 3. Midkine as a Novel Tumor Marker

Numerous studies have demonstrated that MK expression in various malignant tumors is significantly higher than that in normal tissues [53,54,55,56]. Overexpression of MK at both mRNA and protein levels was reported in a variety of human malignancies, such as oral, gastrointestinal, hepatobiliary, lung, thyroid, bladder, cervical, ovarian, and prostate cancer [53,55,56,65,66,67,68,69,70,71,72,73]. Moreover, MK protein expression has been shown to be strongly correlated with poor prognosis in patients with neuroblastomas, astrocytomas, pancreatic head carcinomas, or gastrointestinal stromal tumors [74,75,76,77]. It should be noted that normal tissues of human adults show restricted expression of MK, whereas most carcinoma specimens express MK at significantly high levels in a tissue type-independent manner [53,54,64,78]. In addition to the characteristics of MK expression, because MK protein is a plasma-secreted protein, MK concentrations in blood may increase in patients with malignant diseases. Enzyme-linked immunoassay (EIA) has allowed measurement of MK levels in blood, and increased blood MK concentrations were reported in patients with malignant tumors, including hepatocellular, gastric, and lung carcinoma [79,80]. A series of findings suggests that MK in blood may have potential to become a sensitive and useful tumor marker. Accordingly, Ikematsu *et al*. reported that higher plasma MK concentrations in patients with neuroblastoma were strongly correlated with poor survival [81]. Thus, it is suggested that blood MK concentrations may also become a novel diagnostic marker for predicting prognosis of patients with various cancers described in this section.

### 3.1. Neuroblastoma

Neuroblastoma is the most common extracranial solid tumor in children, comprising between 8% and 10% of all childhood cancers, and accounts for 15% of all childhood cancer deaths, indicating the poor prognosis of many of the tumors [82,83]. A number of genetic and biological features have been recently investigated in an effort to reveal the pathogenesis of neuroblastoma and to identify useful tumor markers. Many prognostic studies have identified several tumor markers associated with overall or disease-free survival, including *MYCN* copy number, tyrosine kinase A (TrkA) expression level, ploidy, and deletion or loss of heterozygosity of chromosome 1p and gain of chromosome 17q [82]. However, because invasive diagnostic procedures, such as a tumor biopsy, are required for these tumor markers [83,84], development of more useful and non-invasive tumor markers is urgently needed to improve the prognosis of patients with neuroblastoma.

Various studies have confirmed that MK is highly expressed in neuroblastoma [74,81,86]. Nakagawa *et al*. first reported that MK mRNA expression was elevated in neuroblastoma specimens at all stages [74]. In agreement with its high mRNA expression, the plasma MK concentrations become elevated with advancing neuroblastoma stages [81]. A higher level of MK was correlated with *MYCN* amplification, low expression of TrkA, diploidy/tetraploidy, and older age, which are known prognostic factors for neuroblastoma, indicating that the elevated plasma MK concentrations is correlated with poor prognostic factors of neuroblastomas. Furthermore, Ikematsu *et al*. also confirmed there was a striking correlation between high plasma MK level and poor prognosis [86]. Analysis for sporadic neuroblastoma cases also showed that the MK level was also remarkably higher than in non-tumor controls, and correlated with the statuses of *MYCN* amplification and stage. A significant correlation was also observed between high plasma MK level and poor prognosis of sporadic neuroblastoma. Taken together, these reports strongly support that plasma MK level is a prognostic factor for neuroblastoma.

### 3.2. Hepatocellular Carcinoma

Hepatocellular carcinoma (HCC) is a common primary cancer of the liver. HCC, endemic to Asia and Africa with a rising incidence in Western countries, is one of the most common and aggressive cancers worldwide [87,88]. The high mortality associated with HCC is ascribed to the difficulty to diagnose at an early stage. Indeed, although HCC patients diagnosed at an early stage and thus receiving curative resection exhibit a significantly improved life prognosis, most symptomatic HCC patients are diagnosed at an advanced stage, precluding their chance for surgical intervention [89,90]. Therefore, early diagnosis has been considered as the most important factor to achieve long-term survival for HCC patients.

Even though α-fetoprotein (AFP) and protein induced by vitamin K absence-2 (PIVKA-II) are one of serologic markers widely used for diagnosing HCC patients, the elevated serum AFP is only observed in about 60% to 70% of HCC patients and, to a lesser extent (33–65%), in patients with smaller HCCs [92]. Moreover, non-specific elevation of serum AFP has been found in 15% to 58% of patients with chronic hepatitis and 11% to 47% of patients with liver cirrhosis [92], indicating the necessity of developing a specific and sensitive HCC biomarkers. Aridome *et al*. first reported that the MK mRNA level was higher in HCC specimens than in the corresponding non-cancerous tissues [67]. By immunohistochemical analysis, high expression of MK was observed in specimens from HCC patients [68]. A highly sensitive EIA for MK revealed that 0.6–0.8 ng/mL of MK was detected in serum samples in the majority of HCC cases, whereas the MK levels in the sera of normal human subjects were low or undetectable [79]. In addition, the overexpression level of MK in HCC with intra-hepatic metastasis was significantly higher than that in HCC without intra-hepatic metastasis [92]. It is noteworthy that a significant increase in serum MK is associated with HCC patients, including those with normal serum AFP concentrations [93]. The measurement of serum MK concentrations allows for distinguishing normal and cirrhosis individuals from HCC patients, including those with normal AFP and small tumors from two independent cohorts. Therefore, the combined score of multiple markers, such as MK and AFP and/or PIVKA-II, may improve the prediction accuracy of identifying HCC patients.

### 3.3. Esophageal Squamous Cell Carcinoma

Esophageal carcinoma is one of the most lethal malignant tumors in the gastrointestinal carcinoma family [94]. Once diagnosed with esophageal squamous cell carcinoma (ESCC), the prognosis is very poor, with a five-year survival rate below 10% [95,96]. Thus, identification of the novel sensitive markers with the use of a relatively non-invasive technique, are needed for detecting the presence of ESCC early. The MK mRNA level is higher in esophageal carcinoma specimens than in the corresponding non-cancerous tissues [67,78]. Likewise, esophageal tumor specimens were positively stained with anti-MK antibody by immunohistochemistry, while surrounding normal esophageal tissues in these specimens were not stained [97]. Additionally, Shimada *et al*. revealed that MK expression by the tumor was significantly associated with high level of serum MK and high serum MK concentrations were associated with tumor size, immunoreactivity, and poor survival [98]. It should be noted that the positive rate of serum MK concentrations was significantly higher than any of conventional markers, such as carcinoembryonic antigen (CEA), SCC antigen (SCC-Ag), and cytokeratin 19 fragment (CYFRA21-1). Multivariate analysis indicated that S-MK is an independent prognostic factor [98]. Since serum concentrations of some types of growth factors are useful to predict treatment response and patients’ outcome in ESCC, serum MK may be a useful prognostic marker for ESCC.

### 3.4. Oral Squamous Cell Carcinoma

Oral cancer is one of the common types of human cancer [99,100]. Because the survival rate for patients with oral cancer still remains poor, improved survival of patients with oral cancer requires better techniques for the prediction of prognosis. Oral squamous cell carcinoma (OSCC), the most common of several types of oral cancers, is associated with bad prognosis. Although previous studies have demonstrated the usefulness of tumor-associated antigens for primary diagnosis of OSCC, no tumor markers have given a precise prediction of prognosis [78,101]. Ota *et al*. provided the direct evidence that serum MK concentrations may have the potential to become a very useful tumor marker in OSCC [66]. Serum MK concentrations were significantly higher in patients with OSCC than in healthy controls. Patients in high serum MK groups showed a significantly lower five-year survival rate compared with patients in low serum MK groups. MK expression in blood and cancer tissues is indicative of a strong relationship with malignant potential, and high expression suggests a bad prognosis. Serum MK concentrations may thus be a useful marker not only for cancer screening but also for predicting prognosis of OSCC patients.

### 3.5. Breast Cancer

Breast cancer is a complex genetic disease characterized by the accumulation of multiple molecular alterations [102]. Although well-established clinicopathological factors show strong overall association with patients’ prognosis and outcome, it has become clear that patients with similar features may show distinct outcomes and vary in their response to therapy [103]. In order to improve the poor prognosis of breast cancer, molecular biomarkers including hormone receptors and human epidermal growth factor receptor 2, are assessed and used in routine clinical practice currently [104]. Although several additional biomarkers are extensively studied, only few biomarkers are useful. Ibusuki *et al*. demonstrated that measuring plasma MK levels in combination with conventional markers provided statistically significant improvement in the diagnosis of breast cancer [105]. Plasma MK levels were abnormally elevated in patients with breast cancer compared to healthy controls. Increased levels of MK were correlated with menopausal status and nuclear grade in primary invasive breast cancer without distant metastasis. In addition, cancer detection rates based on MK levels were higher than those based on three conventional markers including CEA, carbohydrate antigen 15-3 (CA15-3), and Nation Cancer Center-Stomach-439 (NCC-ST-439). Moreover, detection rates of breast cancer using a combination of two conventional tumor markers (CA15-3/CEA, CA15-3/NCC-ST-439, or CEA/NCC-ST-439) with MK was significantly higher than those using combination of three conventional tumor markers. Further clinical validation studies are needed to establish the clinical significance of MK in the plasma of patients with breast cancer.

### 3.6. Pancreatic Cancer

Pancreatic cancer is one of the most aggressive malignancies and has a five-year survival rate of 1–4% [106]. Although surgery is the only curative treatment for patients with pancreatic cancer, only approximately 10–20% of patients have surgically resectable disease at the time of initial presentation, and even in these cases, the five-year survival rate is only 20%. Therefore, development of novel diagnostic methods is urgently needed for the patients to undergo the surgery at the early stage of the disease. Ohhashi *et al*. investigated the feasibility of quantitative analysis of MK mRNA by quantitative real-time RT-PCR (qRT-PCR) as a promising tool for pancreatic cancer diagnosis [107]. Several pancreatic cancer cell lines originating from metastatic lesions, such as KP-3, AsPC-1, SUIT2, and CFPAC1, showed especially high levels of MK mRNA expression. The median value of MK mRNA expression in pancreatic cancer tissue was 5.4-fold higher than those of pancreatic non-neoplastic tissues. These data suggest that quantitative analysis of MK mRNA provides an objective and sensitive evaluation and may be a promising modality for the diagnosis of pancreatic cancer and the prediction of its prognosis. Further investigation on correlation between elevated plasma MK concentrations and prognosis of pancreatic cancer will strengthen the usefulness of MK as a novel prognostic marker.

## 4. Conclusions

**Figure 2:**
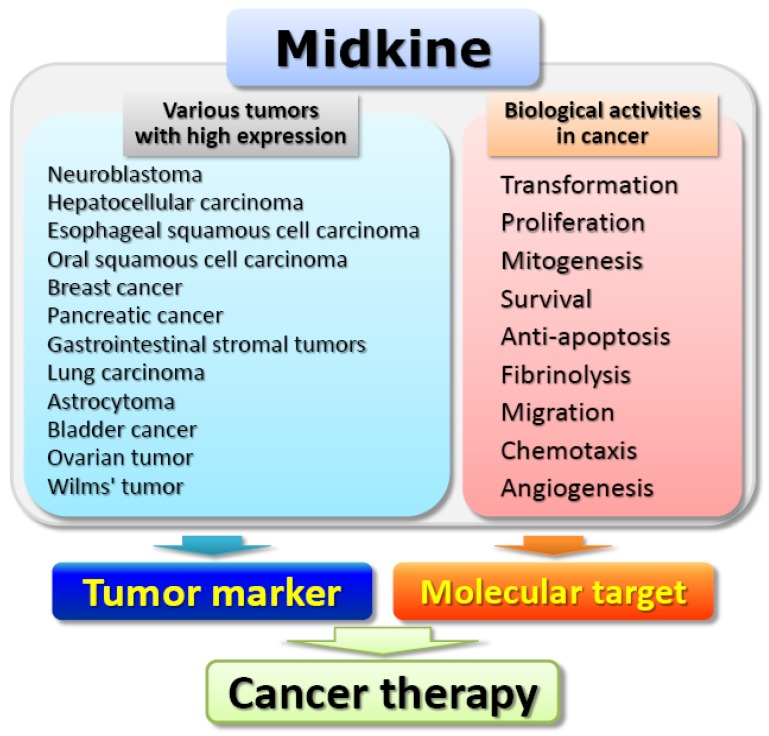
The clinical significance of MK in cancer therapy.

In this review, we described the unique biological features and clinical significance of MK, particularly focusing on the possibility of MK as a novel tumor marker. More additional studies have demonstrated that a truncated form of MK (t-MK), which lacks exon 3 encoding the N-terminus, is also found at both mRNA and protein levels in various tumors, including colon, breast, gastric, liver, and kidney, but not in normal specimens [108,109,110,111,112,113]. Because the frequency of the t-MK expression increases during tumor progression, t-MK may also be utilized as a tumor marker, especially to detect metastatic foci [111,114]. Furthermore, due to its biological significance in carcinogenesis, there is a growing body of evidence that MK can be regarded as a candidate molecular target for therapy against human carcinoma. Indeed, antisense MK oligodeoxyribonucleotides show anti-tumor activity for neurofibroma derived-cells and mouse rectal carcinoma cells [114,115]. Small interfering RNA (siRNA) mediated inhibition of MK expression also has an antitumor effect in prostate and gastric cancer [116,117]. In addition, MK promoter-based conditionally replicative adenovirus therapy for MK high expressing tumors has been attempted recently [118,119,120,121,122,123]. The 5’ regulatory region of the human MK promoter regulates and determines its tumor-specific MK expression. Because of the tumor-specific MK expression, the MK promoter-thymidine kinase gene exhibits a less side effect, indicating that a suicide gene delivery under the control of the MK promoter is a high potential strategy for cancer therapy. Collectively, a series of evidence described above strongly support the possibility of clinical application of MK (Figure 2). Understanding more biological feature and clinical significance of MK may not only bring new insights into the development of a sensitive and specific tumor marker, but may also open up novel therapeutic strategies for a large number of diseases.
